# Molecular mechanisms and targeted interventions for embolic risk in cardiac myxoma: from molecular heterogeneity to clinical translation

**DOI:** 10.3389/fcvm.2026.1884926

**Published:** 2026-07-07

**Authors:** Shichao Guo, Zhiyuan Wang, Yingying Guo, Youwei Zhao

**Affiliations:** Department of Cardiovascular Surgery, The First Hospital of Hebei Medical University, Shijiazhuang, China

**Keywords:** cardiac myxoma, single-cell transcriptome, targeted intervention, tumor embolism, tumor micro environement

## Abstract

Cardiac myxoma (CM), the most common primary cardiac tumor, poses a significant threat to life primarily through embolic complications. While traditional risk assessment relied on clinical and morphological features, the precise molecular determinants of embolism remained poorly understood. Recent breakthroughs in single-cell and spatial transcriptomics have unveiled unprecedented insights into the cellular ecosystem of CM, revealing that embolic propensity is an active process driven by intrinsic tumor cell heterogeneity and a profoundly immunosuppressive microenvironment. This review synthesizes these recent discoveries to establish a novel paradigm: embolism arises from the confluence of a specific PLAT-high tumor subpopulation with dysregulated phosphodiesterase signaling, which impairs cell adhesion, and an M2 macrophage-dominated microenvironment that promotes tumor survival and friability. We critically analyze how these molecular mechanisms can be integrated with existing clinical models to refine embolism prediction. Furthermore, we propose a forward-looking perspective on translating these findings into targeted interventions, such as phosphodiesterase inhibitors and macrophage repolarization strategies, which hold promise as adjunctive therapies to mitigate embolic risk preoperatively and postoperatively, ultimately aiming to improve patient outcomes. This review is the first to systematically integrate the 2024 breakthroughs in single-cell and spatial transcriptomics of CM, establish a novel dual-paradigm of embolic pathogenesis coupling tumor cell intrinsic heterogeneity and immunosuppressive microenvironment crosstalk, and provide a actionable roadmap for clinical translation of molecular-guided risk stratification and targeted therapy.

## 1 Introduction

Cardiac myxoma (CM), accounting for 50%–80% of all primary cardiac tumors, is histologically benign but clinically treacherous due to its potential for embolic events, which occur in 10%–25% of patients and are a notable cause of stroke in young adults (1–3). For decades, the risk of embolism was attributed almost exclusively to macroscopic physical characteristics of the tumor, such as mobility, friability, and anatomical location (1, 4). This perspective, however, failed to fully explain the unpredictable nature of embolism, as patients with similar tumor morphology can exhibit vastly different clinical courses.

The advent of high-resolution molecular profiling technologies, particularly single-cell RNA sequencing (scRNA-seq) and spatial transcriptomics (ST), has catalyzed a paradigm shift. Groundbreaking studies in 2024 have begun to decipher the intricate cellular composition and molecular dialogue within the CM tumor microenvironment (TME) (5–7). These studies highlight that embolism is not a passive, mechanical event but an active process orchestrated by distinct tumor cell subpopulations possessing specific molecular signatures and their dynamic interactions with immune cells.

This review synthesizes 2024 breakthroughs in single-cell and spatial transcriptomics to propose a dual-driver paradigm of CM embolism, coupling tumor cell heterogeneity and immunosuppressive microenvironment crosstalk. We further discuss its translational implications for molecular-guided risk stratification and targeted adjunctive therapies to surgical resection.

## 2 The clinical conundrum of embolic risk stratification

Embolic events in CM patients are diverse, affecting the cerebral (45%–60%), peripheral (20%–30%), and visceral (10%–15%) circulations (8, 9). While clinical scoring systems and meta-analyses have identified age, smoking, atrial fibrillation, tumor morphology, and structural friability as key predictors (10). These models are based on correlative clinical observations and lack a foundation in the biological mechanisms driving embolism. These limitations of morphology-based risk stratification underscore the critical need to integrate molecular insights to achieve truly personalized risk prediction and are most pronounced in the genetically distinct Carney complex-associated CM subtype.

## 3 A New paradigm: molecular mechanisms of embolism

**Figure 1:**
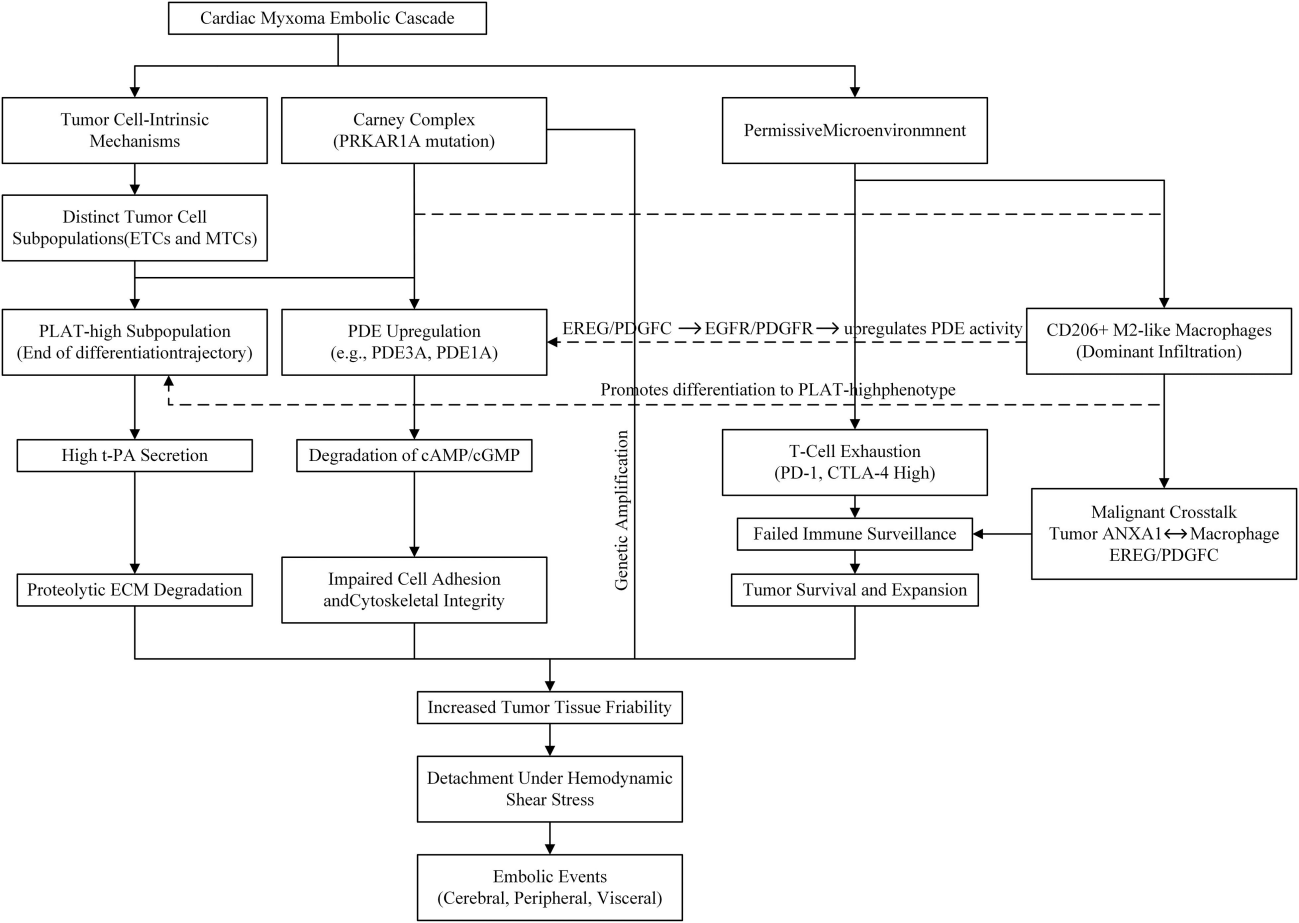
Molecular mechanisms of embolic pathogenesis in cardiac myxoma. ANXA1, annexin A1; cAMP, cyclic adenosine monophosphate; cGMP, cyclic guanosine monophosphate; CTLA-4, cytotoxic T-lymphocyte-associated protein 4; ECM, extracellular matrix; EREG, epiregulin; ETCs, endothelial-like tumor cells; MMP9, matrix metalloproteinase 9; MTCs, mesenchymal stem cell-like tumor cells; PD-1, programmed cell death protein 1; PDE, phosphodiesterase; PDGFC, platelet-derived growth factor C; PLAT, plasminogen activator, tissue type; t-PA, tissue-type plasminogen activator.

CM embolism is not a random mechanical event but a sequential, biologically regulated cascade: impaired tumor cell adhesion → excessive extracellular matrix (ECM) degradation → increased tumor tissue friability → tumor fragment detachment under hemodynamic shear stress → distal embolization in systemic circulation (Figure 1). The central thesis emerging from recent research is that this entire cascade is driven by two synergistic forces: tumor cell-intrinsic molecular abnormalities and a permissive, pro-embolic tumor microenvironment.

### 3.1 Tumor cell-intrinsic mechanisms: the PLAT-high subpopulation and adhesion failure

scRNA-seq analyses have revealed that CMs are composed of heterogeneous cell populations, primarily including endothelial-like tumor cells (ETCs) and mesenchymal stem cell-like tumor cells (MTCs) (6, 11). Crucially, a subpopulation of tumor cells at the end of the differentiation trajectory exhibits high expression of the PLAT gene, which encodes tissue plasminogen activator (t-PA) (5, 12). This subpopulation is hypothesized to be inherently prone to detachment due to t-PA's role in proteolytic degradation of the extracellular matrix (ECM).

Concurrently, a widespread dysregulation in CM cells involves the marked upregulation of phosphodiesterase (PDE) family members (e.g., PDE3A, PDE1A) (5, 13, 14). PDEs degrade cyclic nucleotides (cAMP and cGMP), key second messengers that regulate cell adhesion and cytoskeletal integrity. The overactivity of PDEs likely leads to diminished intracellular cAMP/cGMP levels, thereby weakening cell-cell and cell-ECM interactions. This creates a state of compromised adhesion, making tumor cells vulnerable to detachment under hemodynamic stress.

- Integrated View: The coexistence of a PLAT-high, proteolytically active subpopulation and a global impairment of adhesion signaling via PDE dysregulation creates a potent mechanism for embolism. The former actively dismantles the surrounding matrix, while the latter reduces the cells’ ability to hold on.

### 3.2 The permissive microenvironment: role of M2 macrophages and immune evasion

The TME of CM is not a bystander but an active contributor to embolic risk. Spatial transcriptomic studies have identified a dominant infiltration of CD206+ M2-like macrophages that are spatially proximal to tumor cells (5, 15). These macrophages are not merely present; they engage in a malignant crosstalk with myxoma cells. Macrophage-derived factors such as EREG and PDGFC may bind to receptors on tumor cells, promoting their survival and proliferation (16, 17). In reverse, tumor cell-secreted factors like ANXA1 can reinforce the M2-polarized, pro-tumor state of macrophages (18, 19). This symbiotic relationship fosters a tumor-promoting and immunosuppressive niche.

Furthermore, T-cell function is severely compromised in CM, with abundant exhausted T cells expressing inhibitory receptors like PD-1 and CTLA-4 (20–22). This failure of immune surveillance allows the tumor and its fragile ecosystem to thrive unchecked. The M2 macrophages, through direct interaction and paracrine signaling, may further facilitate the detachment of the adhesion-compromised tumor cells identified in section 3.1.

- Integrated View: The immunosuppressive, M2-dominated TME acts as a catalyst for embolism. It supports the survival and expansion of the fragile tumor cell population and may directly facilitate their dissociation, while a disabled T-cell response fails to eliminate these dangerous subpopulations.

## 4 Carney Complex-associated CM: A genetic amplification of core embolic pathways

Approximately 7%–10% of cardiac myxomas (CMs) occur in patients with Carney complex (CNC), an autosomal dominant syndrome caused by germline *PRKAR1A* inactivating mutations. This hereditary subtype carries a markedly elevated embolic risk (30%–45%, nearly twice that of sporadic CM), alongside earlier onset (20–30 years), 30%–50% multifocality and 20% 10-year recurrence rate (23, 24).

Mechanistically, germline PRKAR1A loss-of-function mutations drive constitutive, ligand-independent PKA activation and sustained cyclic nucleotide elevation. As a homeostatic compensatory response, tumor cells upregulate PDE isoforms (e.g., PDE3A, PDE1A) to degrade excess cAMP/cGMP, but this compensation eventually fails under chronic PKA hyperactivation. The resultant localized cyclic nucleotide depletion at the plasma membrane disrupts AKAP-dependent adhesion complexes, impairing cell adhesion and increasing tumor friability: it further upregulates phosphodiesterase (PDE) activity to exacerbate cell adhesion defects, and promotes enhanced CD206+ M2 macrophage infiltration to create a more pro-friable tumor microenvironment.

Clinically, standard surgery is often insufficient for CNC patients, making this genetically defined population the ideal priority cohort for evaluating the targeted adjuvant therapies proposed later.

## 5 Bridging the Gap: integrating molecular insights into clinical risk prediction

The critical challenge is to connect the molecular mechanisms described above with clinical practice. Current risk models rely on parameters like tumor morphology and atrial fibrillation (25). The new molecular understanding allows us to propose a next-generation, integrated risk assessment framework.

### 5.1 Biomarker discovery

The presence of the PLAT-high subpopulation could be interrogated indirectly by measuring plasma t-PA levels. Similarly, a signature of M2 macrophage infiltration (e.g., soluble CD206, EREG) could be evaluated in serum (5, 26, 27). These circulating biomarkers could provide a real-time, biological readout of embolic risk that complements static imaging features.

### 5.2 Enhanced imaging

Radiomic analysis of cardiac CT or MRI could be developed to identify features correlating with underlying molecular heterogeneity, such as textural patterns indicative of loose ECM or specific perfusion characteristics (28–31).

### 5.3 Future integrated model

A powerful predictive tool would combine the established clinical risk score (e.g., Liu's score) with quantitative data from circulating biomarkers and advanced imaging radiomics. This multi-parametric approach would move risk stratification from correlation to causation. Notably, this integrated model should be separately validated in the CNC population, where traditional clinical risk factors underestimate embolic risk.

## 6 Translational perspectives: from mechanisms to targeted interventions

The elucidated molecular mechanisms open avenues for novel therapeutic interventions, particularly for patients at high embolic risk who are not immediate surgical candidates or for adjuvant therapy to prevent recurrence.

- Targeting Tumor Cell Adhesion: Phosphodiesterase inhibitors (e.g., targeting PDE3A), which are already used in cardiovascular medicine, represent a rational strategy. By elevating cAMP/cGMP levels, these drugs could potentially strengthen cell adhesion and reduce the propensity for detachment. Their repurposing for CM deserves preclinical investigation (32).
- Caveat for Carney Complex Patients: The therapeutic value of PDE inhibitors differs by patient subgroup. For sporadic CM, PDE inhibition elevates cyclic nucleotides to stabilize cell adhesion and reduce embolic risk. However, in Carney complex patients with germline PRKAR1A mutations, constitutive PKA hyperactivation already drives aberrant proliferation. Systemic PDE inhibition may further amplify PKA signaling and exacerbate tumor growth, creating a theoretical “fuel-on-fire” risk. Thus, PDE inhibitor strategies require patient stratification and individualized risk-benefit assessment, with dedicated studies needed to verify safety in Carney complex populations.
- Remodeling the Microenvironment:​ Strategies to reprogram M2 macrophages towards an M1 anti-tumor phenotype (e.g., using CSF1R inhibitors or nanomedicine approaches) could disrupt the pro-embolic niche (33, 34). Similarly, immune checkpoint inhibitors could be explored to reverse T-cell exhaustion, though their application in a benign tumor setting would require careful risk-benefit analysis (35–37).
- Surgical Timing and Adjuvant Therapy: For high-risk patients identified by an integrated model, surgery remains paramount (38–40). However, pre-operative short-term administration of a PDE inhibitor or other targeted agent could theoretically “stabilize” the tumor, reducing perioperative embolic risk. Postoperatively, such agents could be investigated to target minimal residual disease and prevent recurrence (41–43). This approach is particularly critical for CNC patients, who have the highest risk of recurrence and re-embolization.

## 7 Critical synthesis and discussion

This review proposes a unifying dual-driver paradigm for cardiac myxoma embolism: tumor cell-intrinsic defects and an immunosuppressive microenvironment, which form a self-reinforcing vicious cycle (5, 6). *PLAT*-high tumor cells degrade extracellular matrix and recruit M2 macrophages; in turn, M2 macrophages secrete EREG, PDGFC and MMP9 to further exacerbate adhesion impairment and matrix breakdown (5, 15, 16). This cycle is amplified in Carney complex-associated myxomas due to constitutive cAMP-PKA activation (44, 45).

Three key unresolved controversies remain: inconsistent tumor cell subpopulation definitions across single-cell studies, uncertain independent prognostic value of M2 macrophage infiltration, and lack of *in vivo* functional validation for the PDE/cAMP pathway.

We stratify evidence into three levels: clinically validated traditional risk factors (high confidence) (1, 4, 10), mechanistically supported tumor heterogeneity and immune microenvironment features (moderate confidence) (5, 6, 15), and omics-derived hypotheses including *PLAT* subpopulation causality and PDE targeting efficacy (low confidence) (5, 12).

This paradigm explains the limitations of morphology-based risk stratification and calls for integrated clinical-molecular risk models and rigorous functional validation of proposed targeted interventions (45, 46).

## 8 Limitations and future directions

Current research is constrained by the rarity of CM, leading to small sample sizes and a lack of robust *in vivo* models (28, 46). Future work must focus on multi-center collaborations to validate these molecular findings in larger cohorts, with dedicated sub-analyses for the genetically distinct CNC subtype. The development of patient-derived xenograft or organoid models is critical for functional validation of mechanisms and screening of potential therapeutics (45, 47–49). Longitudinal studies are needed to confirm the prognostic value of proposed biomarkers (50, 51).

## 9 Conclusion

The understanding of embolism in cardiac myxoma has evolved from a purely mechanical model to a sophisticated molecular narrative. The interplay between a vulnerable, adhesion-deficient tumor cell subpopulation and a supportive, immunosuppressive microenvironment is central to embolic pathogenesis. Integrating these molecular insights into clinical practice through biomarker-enhanced risk models and exploring mechanism-based targeted therapies hold the promise of transforming the management of CM patients from reactive to proactive, ultimately minimizing the devastating consequences of embolic events.
